# Supplementary material for: Trends in Overall Survival among Patients Treated for Sarcoma at a Large Tertiary Cancer Center between 1986 and 2014
Source: Cancers (Basel). 2023 Jan 14;15(2):514. doi: 10.3390/cancers15020514 (PMC9856368; doi:10.3390/cancers15020514)
Supplement: Supplementary file 1 [file cancers-15-00514-s001.zip › 2023_01_02_Sarcoma_supplement_tables_2-4.pdf]

Supplemental table 2.
Histological subtypes of individuals diagnosed with sarcoma at MCC, 1986-2014.

	N	HR (95% CI)	p-value
**Bone sarcoma subgroups**			5.00E-04†
Osteosarcoma	209 (8.1%)	1.00 (referent)	
Chondrosarcoma	190 (7.4%)	0.59*** (0.44-0.80)	
Ewing sarcoma	128 (5%)	1.19 (0.89-1.59)	
**Soft tissue sarcoma subgroups**			
Leiomyosarcoma	519 (20.2%)	1.22 (0.99-1.51)	
Liposarcoma	467 (18.2%)	0.72** (0.57-0.91)	
Spindle cell sarcoma/NOS	375 (14.6%)	1.47** (1.17-1.86)	
Gastrointestinal stromal tumor	307 (11.9%)	0.70** (0.54-0.90)	
Synovial sarcoma	135 (5.3%)	0.82 (0.61-1.11)	
Rhabdomyosarcoma	50 (1.9%)	1.25 (0.83-1.89)	
Endometrial stromal sarcoma	63 (2.5%)	0.63* (0.42-0.93)	
Other	127 (4.9%)	1.23 (0.92-1.65)	

Abbreviations: HR = hazard ratio; CI = confidence interval
†: Fisher's exact t-test used
* p ≤ 0.05, ** p ≤ 0.01 ***, p ≤ 0.001, **** p ≤ 0.0001

Supplemental Table 3.
Histological subtypes on changes in overall survival over time in individuals diagnosed with sarcoma at MCC, 1986-2014

	1986-1994 aHR[a] (95% CI)	1995-1999 aHR[a] (95% CI)	2000-2005 aHR[a] (95% CI)	2006-2010 aHR[a] (95% CI)	2011-2014 aHR[a] (95% CI)
	N=207	N=208	N=636	N=795	N=724
**Sarcoma type**					
Bone sarcoma	1.00 (referent)	0.96 (0.62-1.47)	0.98 (0.69-1.38)	0.94 (0.66-1.34)	0.58* (0.35-0.94)
STS	1.00 (referent)	0.69** (0.53-0.90)	0.89 (0.72-1.10)	0.80 (0.65-1.00)	0.74* (0.58-0.95)
**Age at Diagnosis (yrs)**					
15-39	1.00 (referent)	0.96 (0.62-1.47)	0.98 (0.69-1.38)	0.94 (0.66-1.34)	0.58* (0.35-0.94)
≥ 40	1.00 (referent)	0.69** (0.53-0.90)	0.89 (0.72-1.10)	0.80 (0.65-1.00)	0.74* (0.58-0.95)
**Ethnicity**					
Non-Hispanic	1.00 (referent)	0.76* (0.60-0.95)	0.95 (0.79-1.15)	0.83 (0.68-1.00)	0.75* (0.60-0.94)
Hispanic	1.00 (referent)	1.01 (0.37-2.72)	0.52 (0.23-1.20)	0.83 (0.37-1.84)	0.49 (0.20-1.21)
Unknown	1.00 (referent)	NE	NE	0.83 (0.15-4.53)	0.63 (0.12-3.33)
**Tobacco use**					
Never	1.00 (referent)	0.67* (0.49-0.91)	0.78 (0.61-1.00)	0.76* (0.59-0.97)	0.58*** (0.42-0.79)
Former user	1.00 (referent)	0.84 (0.51-1.40)	0.91 (0.60-1.38)	0.86 (0.56-1.32)	0.85 (0.53-1.35)
Current	1.00 (referent)	1.02 (0.60-1.72)	1.26 (0.82-1.92)	0.99 (0.64-1.52)	1.32 (0.78-2.23)
Unknown	1.00 (referent)	0.86 (0.43-1.73)	1.55 (0.82-2.95)	2.81* (1.12-7.06)	0.75 (0.36-1.57)
**Stage**					
Localized	1.00 (referent)	0.74* (0.55-1.00)	0.89 (0.70-1.14)	0.84 (0.65-1.08)	0.77 (0.57-1.03)
Metastatic	1.00 (referent)	0.99 (0.69-1.40)	1.11 (0.85-1.46)	1.06 (0.81-1.40)	0.85 (0.61-1.18)
Undefined	1.00 (referent)	NE	NE	NE	NE
Unknown	1.00 (referent)	3.26 (0.20-52.14)	0.68 (0.04-11.42)	NE	5.13 (0.26-100.10)
**First Treatment - Chemotherapy**					
Yes	1.00 (referent)	1.01 (0.71-1.45)	1.14 (0.86-1.51)	1.04 (0.78-1.39)	1.07 (0.77-1.48)
No	1.00 (referent)	0.67** (0.50-0.90)	0.77* (0.61-0.98)	0.73* (0.57-0.94)	0.57*** (0.42-0.76)
**First Treatment - Surgery**					
Yes	1.00 (referent)	0.70** (0.54-0.90)	0.86 (0.70-1.06)	0.85 (0.69-1.05)	0.67** (0.52-0.86)
No	1.00 (referent)	1.84* (1.11-3.05)	1.84** (1.22-2.76)	1.14 (0.75-1.74)	1.22 (0.78-1.90)

Abbreviations: aHR = adjusted hazard ratio; CI = confidence interval; NE = not estimated
[a] All characteristics were included in a single multivariable model to calculate aHR
* $p \leq 0.05$, ** $p \leq 0.01$ ***, $p \leq 0.001$

Supplemental Table 4.
Histological subtypes on overall survival in individuals diagnosed with sarcoma at MCC by time period, 1986-2014

	1986-1994 aHR[a] (95% CI)	1995-1999 aHR[a] (95% CI)	2000-2005 aHR[a] (95% CI)	2006-2010 aHR[a] (95% CI)	2011-2014 aHR[a] (95% CI)
	N=207	N=208	N=636	N=795	N=724
**Sarcoma subgroup**					
Osteosarcoma	1.00 (referent)	1.00 (referent)	1.00 (referent)	1.00 (referent)	1.00 (referent)
Chondrosarcoma	1.41 (0.63-3.19)	1.41 (0.63-3.19)	0.59 (0.22-1.56)	0.48* (0.26-0.90)	0.63 (0.36-1.10)
Ewing sarcoma	2.31* (1.12-4.76)	2.31* (1.12-4.76)	0.86 (0.37-2.03)	0.68 (0.39-1.21)	0.80 (0.46-1.40)
Leiomyosarcoma	2.09* (1.14-3.85)	2.09* (1.14-3.85)	1.49 (0.70-3.20)	0.97 (0.62-1.52)	0.78 (0.52-1.18)
Liposarcoma	1.33 (0.69-2.56)	1.33 (0.69-2.56)	0.81 (0.35-1.84)	0.87 (0.55-1.38)	0.57* (0.36-0.90)
Gastrointestinal stromal tumor	NE	0.78 (0.16-3.76)	0.53** (0.33-0.83)	0.22**** (0.13-0.37)	0.21** (0.08-0.54)
Spindle cell sarcoma/NOS	2.34* (1.22-4.48)	2.34* (1.22-4.48)	2.11 (0.92-4.87)	1.50 (0.94-2.40)	1.05 (0.68-1.64)
Synovial sarcoma	1.28 (0.61-2.66)	1.16 (0.44-3.01)	0.96 (0.56-1.65)	0.68 (0.39-1.20)	0.61 (0.19-1.93)
Rhabdomyosarcoma	1.72 (0.73-4.02)	2.96 (0.34-25.63)	0.42 (0.17-1.03)	0.76 (0.36-1.59)	1.97 (0.63-6.23)
Endometrial stromal sarcoma	1.14 (0.33-4.02)	1.18 (0.35-4.04)	0.26** (0.12-0.59)	0.44* (0.23-0.86)	0.45 (0.11-1.86)
Other	1.47 (0.57-3.77)	1.74 (0.54-5.63)	1.88* (1.14-3.11)	1.15 (0.67-1.96)	1.13 (0.38-3.38)
**Age at Diagnosis (yrs)**					
15-39	1.00 (referent)	1.00 (referent)	1.00 (referent)	1.00 (referent)	1.00 (referent)
≥ 40	2.14** (1.35-3.38)	1.86* (1.09-3.19)	1.47** (1.12-1.92)	1.66*** (1.24-2.22)	2.34*** (1.44-3.81)
**Tobacco use**					
Never	1.00 (referent)	1.00 (referent)	1.00 (referent)	1.00 (referent)	1.00 (referent)
Former user	0.76 (0.48-1.20)	1.31 (0.85-2.03)	1.23 (0.97-1.55)	1.07 (0.84-1.36)	1.38 (0.97-1.96)
Current	0.89 (0.57-1.39)	1.05 (0.64-1.72)	1.34* (1.01-1.76)	1.17 (0.88-1.57)	1.70* (1.07-2.72)
Unknown	0.76 (0.43-1.35)	0.87 (0.49-1.55)	1.31 (0.85-2.01)	2.42* (1.12-5.24)	1.00 (0.54-1.82)
**Spread**					
Localized	1.00 (referent)	1.00 (referent)	1.00 (referent)	1.00 (referent)	1.00 (referent)
Metastatic	2.33**** (1.64-3.31)	1.95** (1.28-2.98)	2.78**** (2.20-3.50)	2.33**** (1.86-2.91)	2.10**** (1.45-3.03)
Undefined	NE	NE	2.25 (0.64-7.89)	2.50 (0.34-18.52)	3.65 (0.83-15.96)
Unknown	0.44 (0.06-3.37)	1.85 (0.63-5.41)	0.75 (0.10-5.80)	NE	1.72 (0.50-5.91)
**First Treatment - Chemotherapy**					
Yes	1.00 (referent)	1.00 (referent)	1.00 (referent)	1.00 (referent)	1.00 (referent)
No	0.96 (0.66-1.39)	0.73 (0.48-1.10)	0.73** (0.58-0.91)	0.58**** (0.46-0.74)	0.56** (0.38-0.82)
**First Treatment - Surgery**					
Yes	1.00 (referent)	1.00 (referent)	1.00 (referent)	1.00 (referent)	1.00 (referent)
No	1.37 (0.87-2.13)	5.01**** (3.01-8.36)	2.94**** (2.24-3.86)	1.87**** (1.40-2.51)	2.58**** (1.76-3.79)

Abbreviations: aHR = adjusted hazard ratio; CI = confidence interval; NE = not estimated
[a] All characteristics were included in a single multivariable model to calculate aHR
* p ≤ 0.05, ** p ≤ 0.01 ***, p ≤ 0.001, **** p ≤ 0.0001
